# Thermal Conductivity and Dynamic Viscosity of Water-Based Al_2_O_3_ and Polyurethane-Nanoencapsulated n-Nonadecane Nanofluids: A Comparative Experimental Study of Mono and Hybrid Formulations

**DOI:** 10.3390/nano16120746

**Published:** 2026-06-15

**Authors:** Semahat Doruk

**Affiliations:** Department of Chemical Engineering, Faculty of Engineering, Çankırı Karatekin University, 18100 Çankırı, Türkiye; sbarlak@karatekin.edu.tr; Tel.: +90-376-218-9500

**Keywords:** hybrid nanofluid, thermal conductivity, dynamic viscosity, nanoencapsulated phase change material, n-nonadecane, Al_2_O_3_ nanofluid

## Abstract

Hybrid nanofluids combining thermally conductive nanoparticles with latent heat-storing nanocapsules have attracted growing interest for near-ambient liquid-based thermal management, yet direct comparisons between mono and hybrid phase-change-material-containing systems on a common experimental basis remain scarce. In this work, water-based mono Al_2_O_3_, mono polyurethane-nanoencapsulated n-nonadecane (PU-NEPCM), and Al_2_O_3_/PU-NEPCM hybrid nanofluids were prepared under identical surfactant, sonication, and dispersion conditions, and their thermal conductivity, dynamic viscosity, and Day-1 colloidal stability were characterized over 298–313 K at total volume fractions of 0.1, 0.3, and 0.5 vol.%, with the hybrids prepared at a 50:50 volumetric ratio. At 0.5 vol.% and 313 K, the hybrid (NFH3) exhibited the highest thermal conductivity enhancement (+8.27%), exceeding the corresponding mono Al_2_O_3_ and mono PU-NEPCM nanofluids by 4.6 and 5.2 percentage points, respectively, while maintaining a moderate viscosity penalty. The hybrid formulations also achieved |ζ| = 32–37 mV, exceeding the conventional electrostatic-stabilization threshold and outperforming both mono families. A two-factor analysis of variance (ANOVA) identified particle concentration as the dominant factor governing both properties (*p* < 0.001), with temperature becoming statistically significant only for the hybrid viscosity (*p* = 0.043). The synergy index varied between 0.85 and 1.43 across the tested conditions—reaching values of 1.20–1.43 for the lowest-loaded hybrid (NFH1)—while the performance index remained close to unity (0.97–1.01). These results identify low-loaded Al_2_O_3_/PU-NEPCM hybrid nanofluids as a balanced and stable candidate for near-ambient liquid-based thermal management applications.

## 1. Introduction

Efficient liquid-based thermal management is a key requirement in many modern engineering systems, including lithium-ion battery cooling, electronics thermal regulation, and compact heat-exchange devices that operate under near-ambient conditions. Conventional heat-transfer fluids such as water, ethylene glycol, and water/glycol mixtures remain widely used because of their availability, chemical stability, and ease of circulation; however, their relatively low thermal conductivity often limits cooling capacity in such applications [1]. Dispersing nanoscale additives into base liquids, the nanofluid approach has therefore been extensively investigated as a route to enhanced thermophysical performance [1,2]. A central practical issue, however, is that thermal conductivity enhancement is typically accompanied by an increase in viscosity, leading to higher pressure drop and pumping-power demand [2,3]. Accordingly, the engineering relevance of a nanofluid is not determined by thermal conductivity alone, but by the balance between heat-transfer gain and viscosity penalty [3].

Among solid nanoparticle additives, Al_2_O_3_ has emerged as one of the most extensively studied candidates for nanofluid applications, owing to its relatively high intrinsic thermal conductivity (typically 30–40 W m^−1^ K^−1^ for bulk Al_2_O_3_) [4], chemical inertness in aqueous media, and ready commercial availability [5]. Aqueous Al_2_O_3_ nanofluids have been shown to provide measurable thermal conductivity enhancement that scales with particle loading [5,6,7] and is generally attributed to effective-medium conduction together with two nanoscale transport mechanisms widely invoked in the literature: Brownian-motion-driven micro-convection [8] and the formation of an interfacial nanolayer of ordered solvent molecules around the particle surfaces [9]. However, the same particle loading that enhances conductivity also raises viscosity through hydrodynamic and particle–particle interactions, thereby imposing a hydraulic penalty that constrains practical implementation [6,7]. In parallel, nanoencapsulated phase change materials (NEPCMs) consisting of a phase-change core enclosed within a thin polymeric or inorganic shell have attracted growing interest as thermally functional additives capable of contributing both sensible and latent heat storage to the carrier fluid [10,11]. The encapsulating shell preserves dispersion integrity by preventing core leakage during melting and protecting the PCM from chemical and mechanical degradation under repeated thermal cycling [10,12]. Paraffinic PCMs such as n-nonadecane ($T_{m}=28–32\;{}^{\circ}C$) are particularly suitable for near-ambient applications, as their melting interval lies within the operating temperature window of lithium-ion batteries, low-temperature electronics, and several building-integrated thermal storage systems [13,14].

The rationale behind hybrid nanofluids is that two or more dispersed components, each with distinct thermophysical functions, can be combined within a single suspension to produce an overall response not achievable with mono-component systems [15,16]. In the Al_2_O_3_/PU-NEPCM combination considered here, the two components are functionally complementary: Al_2_O_3_ enhances thermal conductivity through the mechanisms noted above, whereas PU-NEPCM particles contribute additional thermal storage capacity through the latent heat associated with the solid–liquid transition of the encapsulated n-nonadecane core [16,17]. Hybridization, however, does not in itself guarantee improved performance. The effective response of a hybrid suspension depends on a coupled set of factors: particle concentration, temperature, dispersion quality, and inter-particle interactions between chemically dissimilar dispersed phases that may amplify, attenuate, or even reverse the expected benefit of mixing [17,18]. Because the viscosity penalty also scales with total solid loading, an apparent gain in thermal conductivity may be partially or fully offset by a parallel rise in pumping demand. A robust assessment of hybrid PCM-containing nanofluids therefore requires going beyond absolute conductivity values and explicitly examining the conductivity-viscosity trade-off, the relative importance of operating variables, and any synergistic behavior that may emerge from hybridization [18,19].

Despite the substantial body of work on Al_2_O_3_-based nanofluids [5,6,7], on dispersions containing encapsulated PCMs [10,11,12], and on hybrid suspensions [15,16,17], the literature relevant to PCM-containing hybrid nanofluids exhibits four persistent gaps that limit the interpretation of reported trends: (i) mono and hybrid systems are typically examined under different preparation protocols, base fluids, and temperature windows, so that observed differences cannot be unambiguously attributed to hybridization itself [17,20,21]; (ii) absolute thermal conductivity enhancement is emphasized while the conductivity-viscosity trade-off is rarely quantified in a unified manner [18,19,22]; (iii) hybrid responses are seldom referenced explicitly against the corresponding mono-component systems on a common experimental basis [20,21,22]; and (iv) statistical evaluation of the relative contributions of temperature and concentration is rarely incorporated, limiting the identification of dominant control variables for formulation design.

The present study addresses these four gaps through a single integrated experimental framework. Three water-based nanofluid families: mono Al_2_O_3_, mono PU-NEPCM, and an Al_2_O_3_/PU-NEPCM hybrid prepared at a 50:50 volumetric ratio, were synthesized using a common base fluid, identical surfactant chemistry, and the same two-step preparation and sonication protocol, and were characterized over 298–313 K. This temperature window was selected to encompass the solid–liquid transition of n-nonadecane and to align with the operating envelope of practical near-ambient thermal management duties. Within this framework, five elements support the novelty of the present work: First, a directly comparable thermal conductivity and viscosity dataset spanning mono-oxide, mono-encapsulated-PCM, and oxide/PCM hybrid configurations measured under identical conditions across the phase-transition window of the PCM core has, to the authors’ knowledge, not previously been reported on a unified experimental basis. Second, the conductivity-viscosity trade-off is explicitly addressed through normalized properties together with two indicator-based metrics: a synergy index (SI) quantifying the departure of the hybrid response from purely additive mono-component behavior, and a performance index (PI) quantifying the net practical benefit once the viscosity penalty is taken into account. Third, the dispersion stability of the prepared nanofluids is quantified through Day-1 zeta potential measurements to provide an independent stability baseline for the comparative thermophysical analysis. Fourth, a two-factor ANOVA is used to statistically identify the dominant control variable governing both responses, providing a quantitative basis for formulation design rather than purely descriptive parametric trends. Fifth, the experimentally measured thermal conductivity and viscosity responses are compared against established effective-medium and Arrhenius-type formulations, providing a quantitative reference framework for identifying the non-additive contributions specific to the PCM-containing hybrid system.

## 2. Materials and Methods

### 2.1. Materials

γ-Aluminum oxide nanopowder (γ-Al_2_O_3_; <50 nm by field-emission scanning electron microscopy (TEM); Sigma-Aldrich, Darmstadt, Germany, n-nonadecane (≥99 wt.%,〖*T*〗_*m* = 28–32 °C; Alfa Aesar, Kandel, Germany), toluene-2,4-diisocyanate (TDI, ≥98 wt.%; TCI, (Tokyo Chemical Industry), Tokyo, Japan), diethylenetriamine (DETA, ≥98 wt.%; Merck, Darmstadt, Germany), cyclohexane (≥99 wt.%; Sigma-Aldrich, Germany), and nonylphenol ethoxylate surfactant (NP-10; Sigma-Aldrich, Germany) were used as received. Deionized water (≥18.2 MΩ cm) served as the base fluid. The density of the synthesized PU-NEPCM was determined as 1.19 g cm^−3^ by gas pycnometry (Quantachrome, Boynton Beach, FL, USA), in agreement with our previous report [23]. Component densities used in the volumetric formulation are listed in Table 1.

### 2.2. Preparation of Polyurethane Nanocapsules and Nanofluids

#### 2.2.1. Synthesis of PU-NEPCM Nanocapsules

**Figure 1:**
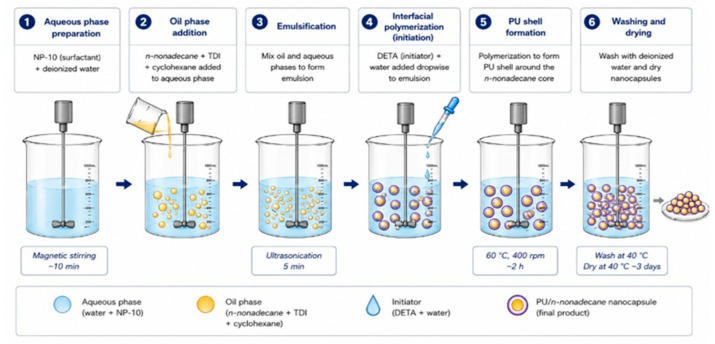
Schematic of the PU-NEPCM nanocapsule synthesis by interfacial polymerization.

Nanocapsules were synthesized by interfacial polymerization in an oil-in-water emulsion following the procedure detailed in our previous work [23]; the overall sequence is summarized in Figure 1. Briefly, an aqueous NP-10 phase was emulsified with an oil phase containing n-nonadecane and TDI in cyclohexane (5 min ultrasonication); DETA was then added dropwise to initiate interfacial polyaddition. Polymerization was carried out at 60 °C and 400 rpm for ~2 h; the resulting capsules were washed with deionized water at 40 °C and dried in a vacuum oven at 40 °C for ~3 days.

#### 2.2.2. Preparation of Nanofluids

Three families were prepared at three total volume fractions (0.1, 0.3, 0.5 vol.%): mono Al_2_O_3_ (NFA), mono PU-NEPCM (NFPU), and Al_2_O_3_/PU-NEPCM hybrid (NFH) at a 50:50 volumetric ratio between the two solid components, giving the nine compositions of Table 2. The 0.1–0.5 vol.% window was selected to span the low-to-moderately loaded regime commonly investigated for aqueous Al_2_O_3_ and PCM-containing nanofluids [6,7], while remaining within the range where the colloidal stability of PU-NEPCM particles can be reliably maintained over the duration of a comparative thermophysical measurement. The required masses were calculated from the densities of Table 1 for a final suspension volume of 100 mL. The base fluid (0.5 wt.% NP-10 in deionized water) was first prepared by magnetic stirring; the required solid components were added, pre-mixed (1000 rpm, 30 min), and probe-ultrasonicated (Sonics Vibra-Cell, 400 W, 20 kHz; 8 s on/2 s off pulsed mode, 60 min total) with a cooling jacket maintaining the suspension near room temperature. All preparation parameters were kept identical across the nine formulations.

### 2.3. Instrumentation and Analysis Methods

#### 2.3.1. Characterization of Polyurethane Nanocapsules

Morphology was examined by field-emission scanning electron microscopy (FE-SEM (ZEISS, Oberkochen, Germany, 300 VP)), with the size distribution extracted from a representative micrograph by automated digital image analysis (watershed segmentation, scikit-image; *n* = 703 particles). Functional groups were identified by attenuated total reflectance–Fourier-transform infrared (ATR–FTIR (Bruker, Ettlingen, Germany)), and thermal behavior was characterized by simultaneous differential scanning calorimetry/thermogravimetric analysis (DSC/TGA (TA Instruments, New Castle, DE, USA, SDT 650)) on 5–10 mg samples at 10 °C min^−1^ over 10–60 °C. The encapsulation ratio was calculated from the latent heats according to Equation (3). The density of the dried PU-NEPCM powder, required for volumetric formulation, was determined by gas pycnometry (Quantachrome, USA) following the procedure of [23].

#### 2.3.2. Thermophysical Property Measurements

Thermal conductivity was measured using a KD2 Pro analyzer equipped with a KS-1 single-needle transient hot-wire sensor (Decagon Devices, Pullman, WA, USA; 60 mm length, 1.3 mm diameter; ±5% accuracy). The KS-1 sensor applies a small heat pulse to the needle and analyses the transient temperature response according to the infinite-line heat-source model; its length-to-diameter ratio satisfies the infinite-line-source assumption, and the device complies with ASTM D5334 and IEEE 442 standards. Measurements were performed with the sample held in a borosilicate glass tube of the same diameter and length as the glycerin-filled verification standard supplied with the instrument, so that the sample-cell geometry reproduced the manufacturer’s validated measurement configuration and the infinite-medium condition required by the hot-wire method was satisfied. The KS-1 needle was inserted vertically (90°) and centered along the tube axis through a hole drilled in the screw cap to match the needle diameter and was fixed in place and sealed with parafilm to ensure reproducible vertical alignment and to suppress evaporation and air-induced convection. The default 1-min read time recommended for low-viscosity liquids was used to minimize the heat added to the sample.

Sample temperature was controlled with a Daihan Scientific, Wonju, Republic of Korea 25 L circulating water bath (working range ambient +5 °C to 100 °C; temperature stability ±0.1 °C); the bath water circulated only within the surrounding jacket and did not contact the sample. Because transient hot-wire measurements require purely conductive heat transfer, particular care was taken to eliminate vibration- and convection-induced errors, following the procedure recommended in the instrument manual. For each reading, the circulation bath was switched off, and the sample was allowed to equilibrate before the probe was inserted; the entire assembly was mounted on a stable, vibration-free bench, and measurements were carried out with the air-conditioning and other laboratory equipment switched off, windows closed, and no operator movement in the room, since the KS-1 sensor is sensitive to air currents and floor-borne vibration. All reported measurements were taken below 313 K (40 °C), well within the ≈50 °C upper limit specified by the manufacturer for accurate KS-1 measurements in aqueous media, above which free convection begins to affect the reading.

At each target temperature (298, 303, 308, 313 K), fifteen readings were collected following this protocol, with the bath re-activated and a waiting interval of about 10 min imposed between successive groups of readings to restore thermal equilibrium and re-homogenize the suspension. Individual readings lying outside ±2σ of the running mean were rejected as outliers; on average, only about one reading per sample was discarded (typically one, at most three of the fifteen, i.e., ≈7%), and the surviving readings were averaged with their standard deviation used as the error bar. The low rejection rate and the small coefficient of variation of the retained readings (≈1% on average across all samples) confirm that the suspensions remained effectively homogeneous over the measurement cycle and that progressive sedimentation did not bias the recorded conductivities (see Supplementary Material, Section S1).

Dynamic viscosity. Dynamic viscosity was measured using an SV-10 sine-wave vibroviscometer (A&D Company, Tokyo, Japan; ±1% accuracy) with the same temperature-control arrangement. The SV-10 determines viscosity from the electrical power required to maintain two immersed sensor plates vibrating at a constant frequency (30 Hz) and a small constant amplitude (≈1 mm); the viscosity is inferred from the vibrational drag on the plates rather than from an imposed shear field, so that neither shear stress nor shear rate is measured independently. The instrument therefore reports a single effective (Newtonian) viscosity and is appropriate for low-viscosity, dilute suspensions; it is not suited to systems approaching the percolation concentration, at which a steep, structure-related rise in viscosity would occur. The low solid loadings investigated here (0.1–0.5 vol.%) lie well below any such regime, and Newtonian behavior was assumed within this dilute, near-ambient window, consistent with previous reports for similar systems [5,23]. Three independent readings per temperature were collected; because the SV-10’s two-decimal-place resolution frequently produced identical replicate readings, the ±1% manufacturer accuracy was used as the error bar. Calibration against the deionized-water reference of Incropera and DeWitt [24] yielded agreement within ±1% across the investigated range.

#### 2.3.3. Dispersion Stability Assessment

Each freshly prepared nanofluid was photographed under controlled lighting (Day 1) and characterized by zeta potential measurements on a Malvern, UK, Panalytical Zetasizer Nano at 25 °C (three readings per sample, no dilution). The conventional |*ζ*| ≥ 30 mV threshold for good electrostatic stabilization [18] was used as a reference benchmark.

### 2.4. Definition of the Synergy and Performance Indices

Two indicator-based metrics were calculated from the measured thermophysical data. The synergy index *SI* quantifies the departure of the hybrid thermal conductivity response from a linear-additive reference constructed from the two corresponding mono-component nanofluids:

(1) $$SI=\frac{(k_{hyb}/k_{bf})-1}{\left[(k_{1}/k_{bf})-1]+[(k_{2}/k_{bf})-1\right]}$$

where $k_{hyb}$ is the thermal conductivity of the hybrid nanofluid, *k*_1_ and *k*_2_ are the thermal conductivities of the corresponding mono-component nanofluids prepared at the same total volume fraction, and $k_{bf}$ is the thermal conductivity of the surfactant-containing base fluid (NF0). *SI* > 1 indicates that the hybrid response exceeds the linear sum of the two mono contributions (true synergistic behavior), *SI* = 1 corresponds to an additive response, and *SI* < 1 indicates a sub-additive response. This enhancement-based formulation is more stringent than ratio-based definitions because it isolates the enhancement contribution from the base fluid baseline [25].

The performance index *PI* quantifies the conductivity-viscosity trade-off:

(2) $$PI=\frac{k_{nf}/k_{bf}}{\mu_{nf}/\mu_{bf}}$$

where $k_{nf}$ and $\mu_{nf}$ are the thermal conductivity and dynamic viscosity of the nanofluid and $k_{bf},\;\mu_{bf}$ are the corresponding base fluid properties. *PI* > 1 indicates a net favorable trade-off, *PI* = 1 a neutral balance, and *PI* < 1 a net unfavorable response. It should be noted that *PI* as defined here captures only the steady-state conductive contribution and viscous dissipation; it does not account for the latent heat storage capacity of the encapsulated n-nonadecane core; *PI* values near unity for PCM-containing systems should therefore be interpreted as a lower bound on practical thermal performance.

The encapsulation ratio of the synthesized PU-NEPCM, used in Section 3.1.3 to assess the efficiency of the polyurethane shell formation around the n-nonadecane core, was calculated as:

(3) $$\eta =\frac{\Delta H_{m,NEPCM}}{\Delta H_{m,\mathrm{n-}nonadecane}}\times 100\%$$

where $\Delta H_{mNEPCM},$ is the latent heat of fusion of the encapsulated PU/n-nonadecane capsules and $\Delta H_{m,\mathrm{n-}nonadecane}\;$ is that of the pure n-nonadecane core.

## 3. Results and Discussion

### 3.1. Characterization of PU-NEPCM Nanocapsules

The synthesized PU-NEPCM particles were characterized prior to their use in the nanofluid formulations to confirm that the obtained capsules were morphologically, chemically, and thermally consistent with the system previously reported by the authors [23].

#### 3.1.1. Morphology and Size Distribution

**Figure 2:**
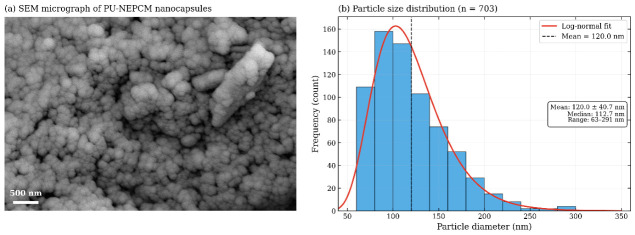
Morphology and size distribution of the synthesized PU-NEPCM nanocapsules.

Figure 2a presents a representative SEM micrograph of the synthesized PU-NEPCM nanocapsules. The capsules exhibit a predominantly spherical morphology without evidence of shell rupture, fracture, or structural collapse, indicating that the polyurethane shell remained intact throughout the synthesis, washing, and drying steps. The particles appear as a dense assembly of individual nano-sized capsules with a tendency to form weakly bound aggregates typical of dry nanocapsule powders. The quantitative size distribution obtained from watershed-based image analysis of the same micrograph (Section 2.3.1) is shown in Figure 2b. The distribution is well-described by a log-normal function and is characterized by a mean diameter of 120.0 ± 40.7 nm (*n* = 703), a median (D50) of 112.7 nm, and a 10th–90th percentile range (D10–D90) of 75.8–173.3 nm; the smallest and largest measured particles fall within 63 and 291 nm, respectively. These values are in close agreement with those previously reported for the same synthesis route, in which a mean diameter of approximately 103 nm and a size range of 55–175 nm were obtained from a manually counted population of more than 200 particles [23], supporting the reproducibility of the interfacial-polymerization procedure across independent batches.

#### 3.1.2. Functional-Group Analysis

**Figure 3:**
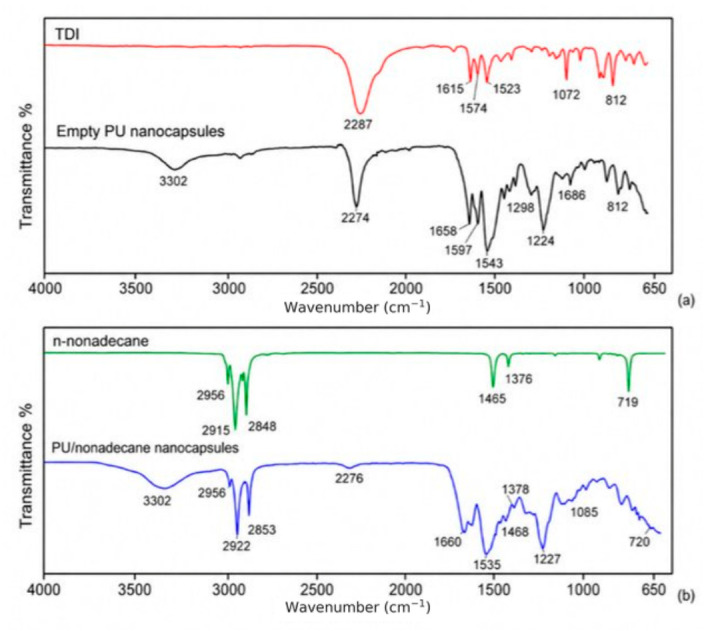
ATR–FTIR spectra confirming polyurethane shell formation and successful encapsulation: (**a**) toluene-2,4-diisocyanate (TDI) and the empty PU nanocapsules; (**b**) pure n-nonadecane and the synthesized PU/n-nonadecane nanocapsules.

Figure 3 shows the ATR–FTIR spectra of pure n-nonadecane, the polyurethane (PU) shell material, and the synthesized PU/n-nonadecane nanocapsules. The spectrum of pure n-nonadecane is dominated by the characteristic C–H stretching vibrations near 2920 and 2850 cm^−1^, together with the C–H bending and rocking modes near 1465 and 720 cm^−1^. The PU spectrum exhibits the N–H stretching band near 3300 cm^−1^, the residual N=C=O stretching of unreacted isocyanate near 2270 cm^−1^, the C=O urethane stretching near 1650 cm^−1^, and the amide-II band near 1540 cm^−1^, in agreement with the expected polyurethane signature. The spectrum of the PU/n-nonadecane nanocapsules clearly contains the characteristic absorption bands of both the n-nonadecane core and the polyurethane shell, confirming that the PCM core was successfully enclosed within the polymeric shell without chemical alteration of either component. The combined spectral fingerprint is consistent with that previously reported for the same system [23], providing further evidence for the chemical reproducibility of the encapsulation procedure.

#### 3.1.3. Thermal Behavior and Encapsulation Efficiency

**Figure 4:**
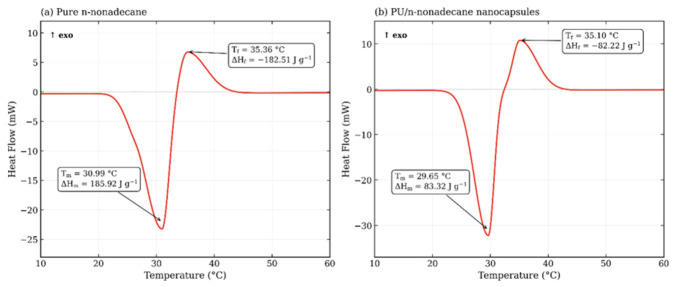
DSC thermograms of (**a**) pure n-nonadecane and (**b**) the synthesized PU/n-nonadecane nanocapsules, showing the endothermic melting and exothermic crystallization peaks.

The thermal behavior of pure n-nonadecane and of the synthesized PU/n-nonadecane nanocapsules was investigated by DSC; representative thermograms are shown in Figure 4. Pure n-nonadecane (Figure 4a) exhibits a sharp endothermic melting peak at $T_{m}=30.99\;{}^{\circ}C$ with a latent heat of fusion of $\Delta H_{m}=185.92\;Jg^{-1}$ and a corresponding exothermic crystallization peak at $T_{f}=35.36\;{}^{\circ}C$ with $\Delta H_{f}=-182.51\;Jg^{-1}$. The PU/n-nonadecane nanocapsules (Figure 4b) display analogous melting and crystallization peaks at ${\;T}_{m}=29.65\;{}^{\circ}C\;\left(\Delta H_{m}=83.32\;Jg^{-1}\right)\;and\;T_{f}=35.10\;{}^{\circ}C\;\left(\Delta H_{f}=-82.22\;Jg^{-1}\right)$, respectively. The persistence of well-resolved melting and crystallization peaks in the encapsulated system confirms that the n-nonadecane core retained its solid–liquid phase-transition functionality after encapsulation within the polyurethane shell.

The encapsulation ratio (*η*) of the PU-NEPCM was evaluated as the ratio of the latent heat of fusion of the encapsulated capsules to that of the pure n-nonadecane core:

(4) $$\eta =\frac{\Delta H_{m,\mathrm{capsule}}}{\Delta H_{m,\mathrm{pure}}}=\frac{83.32}{185.92}=44.8\%.$$

giving *η* = 44.8%. The slight downward shift of the melting temperature (~1.3 °C) observed for the encapsulated system relative to pure n-nonadecane is consistent with confinement-induced suppression of crystalline order within the small PCM domains enclosed by the shell, a phenomenon commonly reported for nanoencapsulated paraffin systems. A comparative summary of the principal characterization parameters obtained in the present work and those previously reported by the authors for the same system is given in Table 3. The near-identical encapsulation ratios (44.8% versus 44.7%) provide strong evidence for the methodological reproducibility of the interfacial-polymerization procedure across two independent preparations carried out nearly a decade apart.

### 3.2. Dispersion Stability

**Figure 5:**
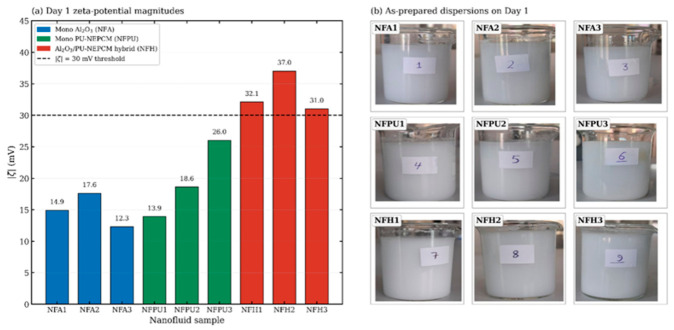
Day-1 dispersion stability assessment of the prepared nanofluids.

The colloidal stability of the prepared nanofluids was assessed immediately after the completion of the ultrasonication step (Day 1) through visual inspection of the dispersion state and through quantitative zeta potential measurements. Figure 5 summarizes both qualitative and quantitative stability indicators across the nine investigated formulations.

Visually, all nine nanofluids appeared as homogeneous, opaque-to-milky white dispersions with no observable macroscopic sedimentation or phase separation within the time scale of the subsequent thermophysical measurements (Figure 5b). This homogeneous appearance was retained throughout the property characterization protocol, supporting the interpretation that the Day-1 measurements reflect the as-prepared dispersion state.

Quantitatively, the measured zeta potential values (Figure 5a) revealed a systematic dependence on both formulation type and total volume fraction. The mono Al_2_O_3_ nanofluids (NFA1–NFA3) exhibited |*ζ*| values in the range of 12.3–17.6 mV, and the mono PU-NEPCM nanofluids (NFPU1–NFPU3) showed a moderate increase from 13.9 mV at 0.1 vol.% to 26.0 mV at 0.5 vol.%, reflecting an enhanced electrostatic contribution from the NP-10-stabilized polymer surface at higher particle loadings. Notably, the hybrid formulations (NFH1–NFH3) yielded substantially higher zeta potential magnitudes of 31.0–37.0 mV, exceeding the conventionally accepted |*ζ*| ≥ 30 mV threshold for good electrostatic stabilization against aggregation [18]. Two implications follow: First, the hybrid dispersions are colloidally well-stabilized at the time of measurement, which is essential for the interpretability of the subsequent thermophysical comparisons. Second, and more unexpectedly, hybridization itself produced a measurable enhancement in colloidal stability beyond that of either mono-component system at the same total volume fraction.

The origin of this hybrid-induced colloidal stabilization can be attributed to the co-presence of two chemically and structurally dissimilar dispersed phases—a metal oxide (Al_2_O_3_) and a polymer-shelled PCM (PU-NEPCM)—whose combination is not a simple average of the two mono behaviors. It should first be emphasized that the two mono systems are not strongly aggregating suspensions that the hybrid somehow rescues; rather, both mono families are already kinetically dispersed by the NP-10 surfactant but carry only a modest electrostatic charge (|*ζ*| = 12.3–17.6 mV for NFA and 13.9–26.0 mV for NFPU), placing them in the weakly stabilized regime. The hybrid combination raises |ζ| above the conventional 30 mV threshold through two cooperative effects: First, the two surfaces differ in their charging behavior: the γ-Al_2_O_3_ surface develops its charge through the protonation/deprotonation of amphoteric surface hydroxyl groups, whereas the PU-NEPCM surface acquires charge mainly through the adsorbed NP-10 layer on the polyurethane shell. When both surfaces are present, the surfactant partitions between two distinct surface chemistries and the suspension carries a more heterogeneous and, in net magnitude, larger charge population than either mono system, raising the measured zeta potential. Second, the marked size and density contrast between the small, dense Al_2_O_3_ nanoparticles (<50 nm; 3.95 g cm^−3^) and the larger, lighter PU-NEPCM capsules (≈120 nm; 1.19 g cm^−3^) introduces a steric/geometric contribution: the smaller oxide particles can occupy the interstitial regions between the larger capsules and hinder their close approach, adding a steric barrier to the electrostatic repulsion and further suppressing aggregation. The combination of an enhanced electrostatic charge and this additional steric hindrance provides a consistent explanation for why the hybrid suspensions are more stable than either mono-component system at the same total volume fraction, rather than merely intermediate between them. This hybrid-induced stabilization represents an additional functional benefit of the Al_2_O_3_/PU-NEPCM combination beyond the thermal conductivity considerations discussed in the subsequent sections.

It should be noted that the present study focuses on the short-term dispersion behavior relevant to the thermophysical-property measurement protocol. The systematic evaluation of long-term colloidal stability, including time-resolved zeta potential evolution, dynamic-light-scattering aggregation kinetics, and sedimentation rate analysis, falls beyond the scope of this work and is reserved for a dedicated future investigation. The Day-1 zeta potential values, together with the visual homogeneity records, nevertheless provide a consistent stability baseline against which the mono and hybrid formulations were characterized under directly comparable conditions.

Because the encapsulated n-nonadecane core repeatedly melts and freezes within the operating temperature window, repeated thermal cycling could in principle influence the long-term dispersion behavior through volume changes of the core and gradual reorganization of the adsorbed surfactant layer; assessing whether such thermal cycling effects alter the colloidal stability of the present formulations is an important direction for the future investigation noted above.

### 3.3. Thermal Conductivity

**Figure 6:**
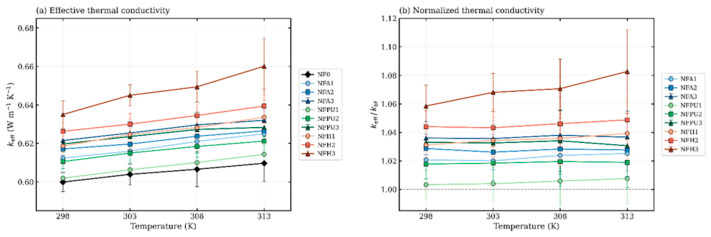
(**a**) Effective thermal conductivity and (**b**) thermal conductivity enhancement ratio of the prepared nanofluids as a function of temperature. Error bars represent ±1 SD.

Figure 6 presents the effective thermal conductivity $k_{eff}$ (panel a) and the conductivity enhancement ratio $k_{eff}/k_{bf}$ (panel b) of all nine nanofluids and the surfactant-containing base fluid (NF0) over the investigated temperature range of 298–313 K. Two general trends are evident across all formulations: (i) $k_{eff}$ increases monotonically with temperature, in line with the temperature dependence of the base fluid and the expected Brownian-motion-assisted heat transport at higher temperatures [8,26]; and (ii) at any fixed temperature, $k_{eff}$ increases with the total solid volume fraction. The enhancement relative to the base fluid is, however, strongly composition dependent: at the maximum loading investigated (0.5 vol.%) and at 313 K, NFA3 (mono Al_2_O_3_) yielded $k_{eff}/k_{bf}=\;1.037\;$ (a 3.66% enhancement), NFPU3 (mono PU-NEPCM) yielded 1.031 (3.06%), and the hybrid NFH3 yielded 1.083 (8.27%). The hybrid response therefore exceeded both mono-component systems by approximately 4.6 and 5.2 percentage points, respectively.

The contrasting behavior of the two mono families reflects their different heat-transport mechanisms. Aluminum oxide nanoparticles contribute primarily through their intrinsically high bulk thermal conductivity (γ-Al_2_O_3_ ≈ 30–40 W m^−1^ K^−1^), so that even at low dilution (0.5 vol.%) they provide a measurable enhancement via the percolative formation of locally conductive paths and interfacial-layer ordering of the surrounding water molecules around the particle surfaces [5,6,7,9]. PU-NEPCM particles, in contrast, possess a low bulk thermal conductivity (~0.25 W m^−1^ K^−1^ as reported in our earlier study [23]) and would, on the basis of pure conductive considerations, be expected to slightly decrease the effective conductivity of the suspension. The fact that all NFPU samples nevertheless showed a positive enhancement (0.76–3.06% at 313 K) indicates that additional, non-conductive mechanisms are at play, most notably (i) the latent heat absorption/release of the encapsulated n-nonadecane core within the phase-transition window (*T*_*m* ≈ 30 °C), which augments the apparent transient heat-flux response sensed by the KS-1 hot-wire probe [12,14], and (ii) Brownian agitation of the nanocapsules, which generates a micro-convective contribution to heat transport [8].

The contrasting size scales of the two dispersed phases warrant a brief comment in relation to the conventional expectation that, for a given material, a smaller particle size favors higher thermal conductivity through the larger specific surface area available for interfacial heat transport and Brownian-motion-driven micro-convection. In the present hybrid system, this expectation cannot be applied directly, because the two constituents differ simultaneously in size and in intrinsic transport mechanism: the γ-Al_2_O_3_ nanoparticles (<50 nm) are considerably smaller than the PU-NEPCM nanocapsules (mean diameter 120.0 nm, Section 3.1.1), yet they also possess a far higher intrinsic conductivity (≈30 versus ≈0.25 W m^−1^ K^−1^). Consequently, the measured conductivity ranking is not governed by particle size as an independent variable but by the dominant heat-transport pathway of each phase. For the smaller Al_2_O_3_ particles, the high specific surface area and high intrinsic conductivity act in the same direction, reinforcing the effective-medium, Brownian, and interfacial-nanolayer contributions; for the larger PU-NEPCM capsules, by contrast, the enhancement originates almost entirely from the latent heat exchange of the encapsulated n-nonadecane core rather than from any size-related conduction effect. The smaller particle size of Al_2_O_3_ therefore contributes to its larger conductive role, but in this hybrid system, the size effect is convoluted with intrinsic conductivity and latent heat contributions and cannot be isolated as a single controlling factor.

The Maxwell effective-medium prediction for non-interacting spherical inclusions provides a useful baseline for assessing the magnitude of these anomalous contributions [4]:

(5) $$\frac{k_{eff}}{k_{bf}}=\frac{k_{p}+2k_{bf}+2\phi (k_{p}-k_{bf})}{k_{p}+2k_{bf}-\phi (k_{p}-k_{bf})}$$

where $k_{p}$ is the conductivity of the dispersed phase and *φ* its volume fraction. Substituting $k_{p}\approx 30\;Wm^{-1}K^{-1}$ for Al_2_O_3_ and $k_{bf}\approx 0.61\;Wm^{-1}K^{-1}$ at 313 K, Equation (4) predicts $\frac{k_{eff}}{k_{bf}}\approx 1.014$ for NFA3 (a 1.4% enhancement), whereas the measured value is 1.037 (3.66%). The measured enhancement therefore exceeds the Maxwell prediction by approximately 2.3 percentage points, consistent with the “anomalous” thermal conductivity enhancement widely reported for low-loaded oxide nanofluids and attributed to Brownian-motion-driven micro-convection and to the formation of an interfacial nanolayer of ordered solvent molecules around the nanoparticles [26,27]. For NFPU3, the Maxwell model based on the bulk PU-NEPCM conductivity predicts a slight decrease (~−0.4%), whereas the measured value is +3.06%; this much larger deviation confirms that the latent heat contribution is the dominant enhancement mechanism for the encapsulated-PCM system.

The substantially larger enhancement of the hybrid formulations cannot be explained by a simple linear combination of the individual mono-component contributions. Evaluating the synergy index from Equation (1) at 313 K and 0.5 vol.% total loading gives *SI* = (1.083 − 1)/[(1.037 − 1) + (1.031 − 1)] = 1.22, indicating that the hybrid response exceeds the linear sum of the two mono contributions by ~22%. The probable origin of this synergy is the coupling of two complementary mechanisms within the hybrid suspension: thermally conductive Al_2_O_3_ paths efficiently transport the heat released or absorbed by the n-nonadecane phase transition within the encapsulated PU-NEPCM particles, while Brownian agitation of the smaller Al_2_O_3_ nanoparticles continuously perturbs the local thermal field around the larger PU-NEPCM capsules, enhancing the effective coupling between sensible and latent heat-transport pathways [15,16,17,27]. A more detailed treatment of the synergy index across temperature and concentration is presented in Section 3.6.

Finally, the temperature slope of $k_{eff}/k_{bf}$ is itself informative. For the mono Al_2_O_3_ family, the enhancement ratio is nearly temperature-independent over the investigated window, consistent with a temperature-insensitive Brownian/interfacial-layer mechanism. For the hybrid family, however, the enhancement ratio increases noticeably with temperature, from ~5.9% at 298 K to ~8.3% at 313 K—a trend that tracks the melting interval of n-nonadecane and supports the interpretation that latent heat-driven contributions become progressively more relevant as the operating temperature traverses the phase-transition window.

### 3.4. Dynamic Viscosity

**Figure 7:**
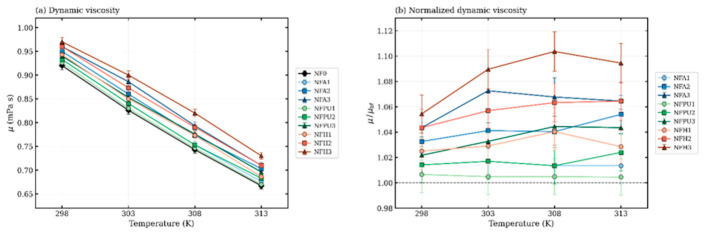
(**a**) Dynamic viscosity, (**b**) Normalized dynamic viscosity.

Figure 7 presents the dynamic viscosity *μ* (panel a) and the normalized viscosity ratio *μ*/*μ*_*bf* (panel b) of all nanofluids over the investigated temperature range. As expected from the temperature dependence of water and dilute aqueous suspensions, all formulations exhibited a monotonic decrease in *μ* with increasing temperature, in line with the thermally activated viscous-flow behavior of the base fluid. At any fixed temperature, *μ* increased with the total solid volume fraction across all three families, consistent with the expectation that dispersed phases hinder momentum transport between adjacent fluid layers and elevate the effective viscosity [2,3].

At the maximum loading investigated (0.5 vol.%) and at 313 K, the measured viscosity ratios were *μ*/*μ*_*bf* = 1.064 for NFA3 (6.45% increase), 1.043 for NFPU3 (4.35%), and 1.094 for NFH3 (9.45%). The hybrid formulation therefore showed the largest viscosity penalty among the three families, exceeding the mono Al_2_O_3_ and mono PU-NEPCM systems by approximately 3.0 and 5.1 percentage points, respectively, at otherwise identical loading and temperature. The relative ordering NFH > NFA > NFPU was preserved across all investigated temperatures and concentrations. At 298 K, the corresponding viscosity increases were comparatively smaller (NFA3: 4.35%, NFPU3: 2.17%, NFH3: 5.43%), reflecting the reduced relative effect of dispersed particles on viscosity at lower temperatures, where the base fluid viscosity itself is higher [2].

The lower viscosity penalty of the NFPU family relative to NFA at otherwise identical loading is consistent with the lower particle–fluid hydrodynamic friction expected for polymer-shelled, lower-density (1.19 g cm^−3^) capsules compared with the denser γ-Al_2_O_3_ nanoparticles (3.95 g cm^−3^). The substantially larger viscosity increase of the hybrid system, in turn, indicates that the simultaneous presence of two chemically and morphologically distinct dispersed phases produces a stronger hydrodynamic-interaction contribution than would be expected from a simple linear combination of the two mono components [28].

To provide a compact, predictive description of the experimental viscosity data and to enable comparison across families, a two-factor correlation was fitted to each family in the form previously used in our earlier study of EG-based PU-NEPCM nanofluids [23]:

(6) $$\mu \left(T,\phi \right)=\left(1+C\phi +D\phi^{2}\right)\;A_{0}\;\exp\left(\frac{B_{0}}{T}\right)$$

Here, *A*_0_ and *B*_0_ describe the temperature dependence of the base fluid viscosity (Arrhenius-type form), and the polynomial term (1 + *Cφ* + *Dφ*^2^) captures the concentration dependence of the relative viscosity at fixed temperature. The fitted coefficients are summarized in Table 4. The coefficient of determination exceeded 0.993 in all three cases, with maximum deviations between fitted and measured values below 1.6%, comparable to the manufacturer-stated ±1% accuracy of the SV-10 vibroviscometer.

Several physical interpretations follow from the fitted coefficients. The temperature parameter *B*_0_ is similar across the three families (1874–1946 K), corresponding to an apparent flow activation energy of ~15.6–16.2 kJ mol^−1^ and indicating that the base fluid sets the dominant temperature dependence, as expected for low-loaded suspensions. The linear concentration coefficient *C* shows the most informative trend: *C*_NFPU = +0.039 corresponds to the smallest concentration sensitivity, *C*_NFA = +0.155 is intermediate, and *C*_NFH = +0.232 is the largest. Notably, *C*_NFH exceeds the sum of the two mono coefficients (*C*_NFA + *C*_NFPU ≈ 0.194) by approximately 20%, confirming that the hybrid viscosity response contains an additional non-additive contribution beyond a simple linear average of the two mono behaviors [2,18].

The negative quadratic coefficient *D*_NFH = −0.153 indicates a gradual saturation of the concentration-induced viscosity increase between 0.3 and 0.5 vol.%, rather than continued steepening; a similar but weaker trend is observed for NFA (*D* = −0.069). This sub-linear behavior is consistent with the onset of inter-particle hindrance effects at the upper end of the investigated concentration range, and is also visually consistent with the slight curvature visible in the *μ*/*μ*_*bf* profiles of Figure 7b for NFA3 and NFH3 at higher temperatures. Equation (5), together with the coefficients of Table 4, therefore provides a compact engineering correlation that captures both the Arrhenius-like temperature dependence and the non-additive hybrid concentration behavior within the investigated parameter window, complementing recent efforts to develop scaling-based correlations for the transport properties of hybrid nanofluids [29].

### 3.5. Statistical Analysis of the Main Thermophysical Responses

A two-factor analysis of variance (ANOVA) was performed on the normalized thermal conductivity ($k_{eff}/k_{bf}$) and normalized dynamic viscosity (*μ*/*μ*_*bf*) of each of the three nanofluid families, using temperature (four levels: 298, 303, 308, 313 K) and total volume fraction (three levels: 0.1, 0.3, 0.5 vol.%) as the independent factors. The aim was to quantify, on a common statistical basis, the relative influence of temperature and concentration on the two thermophysical responses, and to verify whether the qualitative trends discussed in the preceding sections (and the non-additive hybrid behavior captured by the viscosity correlation) are reflected in a formal significance test. The results are summarized in Table 5.

Three principal observations emerge from Table 5. First, particle concentration was the statistically dominant factor for both $k_{eff}/k_{bf}$ and $\mu_{eff}/\mu_{bf}$ across all three families, with *p*-values below 0.001 in five of the six analyses and below 0.005 in the sixth. This formally confirms the observation made qualitatively from Figure 6 and Figure 7 that concentration controls the thermophysical response of the investigated nanofluids more strongly than temperature within the near-ambient range examined here, in line with prior reports for dilute oxide and PCM-containing nanofluids [5,12].

Second, temperature was not statistically significant (*p* > 0.05) for either response in the NFA and NFPU families, indicating that the normalized properties of the two mono systems do not change appreciably over the 298–313 K window and that the thermophysical enhancement they provide can be regarded—at the resolution of the present measurements—as essentially temperature-independent. This is consistent with the near-flat temperature trends of $k_{eff}/k_{bf}$ for the NFA and NFPU families in Figure 6b and with the small temperature variation of the viscosity coefficient *B*_0_ in Table 4.

Third, and most importantly, the temperature effect on $\mu_{eff}/\mu_{bf}$ was statistically significant for the hybrid family only (*p* = 0.043 for NFH). This is consistent with the larger temperature coefficient observed for NFH in the viscosity correlation (Equation (5), Table 4) and with the steeper temperature dependence of the NFH curves in Figure 7b. The combination of hybrid composition and the presence of an encapsulated PCM core that traverses its phase-transition interval within the investigated temperature range (Section 3.1.3) is the most plausible origin of this additional temperature sensitivity, since the solid-to-liquid transition of the n-nonadecane core modifies the effective deformability and interaction of the dispersed PU-NEPCM particles in a way that is absent in the all-solid NFA system and only weakly present in the unmixed NFPU system. The *p*-value for temperature in the NFH thermal conductivity ANOVA (*p* = 0.059), although marginally above the conventional 0.05 threshold, is notably lower than the corresponding values for NFA (*p* = 0.119) and NFPU (*p* = 0.512), supporting the same picture from a different observable.

Taken together, the ANOVA results provide independent statistical support for two of the central interpretations of this study: (i) the dominance of particle concentration over temperature as a control variable for the normalized thermophysical response of low-loaded mono and hybrid nanofluids in the near-ambient range, and (ii) the emergence of a non-trivial temperature sensitivity in the hybrid system consistent with the phase-transition contribution of the encapsulated PCM core. The *R*^2^ values of all six fits exceed 88%, confirming that temperature and concentration together explain the dominant share of the observed variability in both responses.

### 3.6. Synergy and Performance Indices

**Figure 8:**
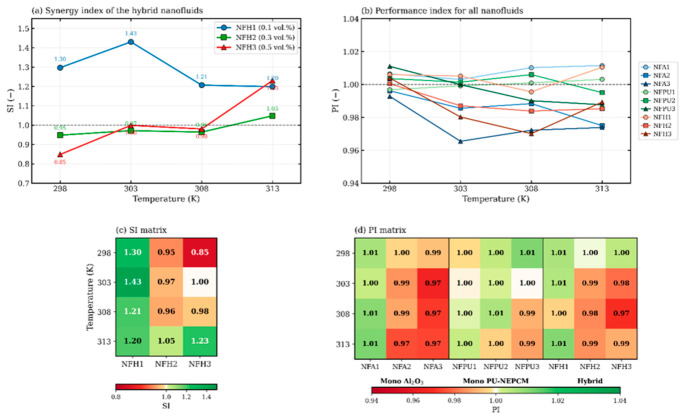
Synergy and performance assessment of the prepared nanofluids.

Figure 8 summarizes the synergy index *SI* and the performance index *PI* of the prepared nanofluids, calculated from Equations (1) and (2) using the measured thermophysical properties at each investigated temperature. The line plots (panels a, b) show the temperature dependence of the two indices, and the heatmap matrices (panels c, d) provide a compact display of all individual values for direct comparison across formulations and temperatures.

The SI values shown in Figure 8a,c reveal a markedly concentration- and temperature-dependent behavior. NFH1 (0.1 vol.% total loading) exhibited a consistently strong synergistic response, with SI between 1.20 and 1.43 across the entire investigated temperature window and a maximum of 1.43 at 303 K. This indicates that at low total loading the hybrid thermal conductivity enhancement exceeded the linear sum of the two mono-component enhancements by 20–43%, providing clear evidence of synergistic coupling between the Al_2_O_3_ and PU-NEPCM constituents. NFH2 (0.3 vol.%) showed essentially additive behavior, with SI values clustered close to unity (0.95–1.05). NFH3 (0.5 vol.%), in contrast, displayed a pronounced temperature-dependent crossover: starting from a sub-additive value of *SI* = 0.85 at 298 K, it remained below or near unity up to 308 K, and then increased sharply to *SI* = 1.23 at 313 K.

This crossover is one of the most informative observations of the present study because it directly tracks the phase-transition window of the encapsulated n-nonadecane core. At sub-melting temperatures, the encapsulated PCM remains in the solid state and contributes only its sensible-heat conductivity to the suspension, which is insufficient to compensate for the inter-component hindrance at high loadings; once the PCM transitions to the liquid state across 303–308 K, its effective participation in heat transport increases, and the cross-component coupling identified in Section 3.3 emerges as a clear synergistic contribution at 313 K. The fact that this crossover is most visible at the highest loading (NFH3), where the absolute PCM volume is largest, supports this interpretation. The opposite ordering at 298 K—where the smallest hybrid (NFH1) shows the strongest synergy and the largest hybrid (NFH3) the weakest—further supports a mechanism in which low loadings favor cross-component coupling through Brownian micro-mixing, whereas high loadings without PCM melting are limited by inter-particle hindrance. This loading-dependent synergy behavior is consistent with the framework recently summarized for hybrid nanofluid systems in [30].

It is worth clarifying whether this loading-dependent synergy reflects differences in dispersion state among the three hybrids, as opposed to a difference in the underlying coupling mechanism. The Day-1 zeta potential data argue against a dispersion-quality explanation: all three hybrid formulations are comparably well stabilized at the time of measurement, with |*ζ*| = 32.1–37.0 mV (Table S3.1), each exceeding the conventional |*ζ*| ≥ 30 mV threshold for good electrostatic stabilization. Since NFH1, NFH2, and NFH3 are all well dispersed, the pronounced contrast in their synergy indices cannot be attributed to one hybrid being better or more poorly dispersed than another. The loading dependence instead arises from a shift in the balance between two competing contributions. At the lowest loading (NFH1, 0.1 vol.%), the dispersed phases are well separated, and Brownian-driven micro-mixing of the small Al_2_O_3_ nanoparticles couples efficiently to the latent heat exchange of the PU-NEPCM capsules, yielding a strong and temperature-robust synergy (*SI* = 1.20–1.43). As the loading increases, inter-particle hindrance progressively counteracts this coupling while the encapsulated core remains solid, so that NFH3 is sub-additive below the melting interval (*SI* = 0.85 at 298 K). Once the temperature traverses the n-nonadecane transition (≈30 °C), the latent contribution is activated, and the cross-component coupling re-emerges, producing the sharp rise to *SI* = 1.23 at 313 K. The loading-dependent synergy is therefore governed by the competition between Brownian micro-mixing and inter-particle hindrance, modulated by the phase state of the encapsulated PCM, rather than by differences in colloidal dispersion among the hybrids.

In contrast to *SI*, which isolates the synergistic component of the thermal conductivity response, the performance index *PI* incorporates the simultaneous viscosity penalty and therefore provides a more direct engineering assessment of the conductivity-viscosity trade-off. As shown in Figure 8b,d, the calculated *PI* values fall within the narrow band of 0.97–1.01 across all nine formulations and four temperatures, indicating that the thermal conductivity enhancement and the viscosity penalty are of comparable magnitude in the present low-loaded systems. Three observations are particularly noteworthy: First, NFH1 maintained PI ≥ 1.00 across the entire temperature range, making it the only formulation in the dataset that simultaneously combines a clearly synergistic thermal response (*SI* = 1.20–1.43) and a non-degraded conductivity-to-viscosity balance. From a practical viewpoint, NFH1 therefore emerges as the most balanced candidate within the investigated parameter window. Second, mono Al_2_O_3_ at 0.3 and 0.5 vol.% (NFA2, NFA3) showed the lowest *PI* values (down to 0.97 at 308–313 K), reflecting the comparatively steep viscosity increase of the denser oxide nanoparticles noted in Section 3.4 from the larger concentration coefficient C of Equation (5). Third, the *PI* values of NFH2 and NFH3 also dropped slightly below unity at higher temperatures (~0.97–0.99), driven by the larger viscosity penalty of the hybrid suspensions at elevated loading.

Two further points clarify why most performance-index values fall marginally below unity and how the conductivity-viscosity trade-off may be improved, and two factors determine whether the balance is favorable: First, the present *PI* definition (Equation (2)) accounts only for the steady-state conductive gain and the viscous penalty and does not credit the latent heat storage capacity of the encapsulated n-nonadecane core; for the PCM-containing families (NFPU, NFH), the reported PI values therefore represent a conservative lower bound on the true thermal performance, most markedly across the phase-transition window where the latent contribution is active. The fact that the NFPU and NFH formulations nevertheless reached *PI* ≈ 1, despite carrying a built-in latent heat reservoir that is not reflected in the $k_{eff}$ measurement, implies that their effective energy-handling capacity exceeds what *PI* alone suggests—consistent with the temperature-dependent rise in *SI* at higher loading discussed above. Conversely, the all-solid NFA system has no latent heat reservoir, and its *PI* values therefore directly represent its full thermal-rheological performance. Second, the trade-off is most favorable at low loading: NFH1 is the only formulation that maintains *PI* ≥ 1.00 at every temperature while also exhibiting strong synergy (*SI* = 1.20–1.43), because at low loading the conductivity gain from hybrid coupling is realized without the steep viscosity rise that accompanies higher solid fractions, as quantified by the large concentration coefficient C of the hybrid viscosity correlation (Table 4). From a practical standpoint, the conductivity-viscosity trade-off is therefore best resolved by adopting a low-loaded hybrid (rather than mono) formulation and, where the operating range spans the PCM melting interval, by exploiting the latent heat contribution that the steady-state PI does not capture. The incorporation of this latent heat term into a unified performance metric is identified as a direction for future work in the Conclusion.

Taken together, the SI and PI analyses indicate that hybrid synergy is real but condition-dependent, most pronounced at low total loading (NFH1) and emerging at high loading only above the PCM melting interval (NFH3 at 313 K), while the conductivity-viscosity balance remains near unity across all systems, with NFH1 providing the most favorable combination of synergy and trade-off within the investigated parameter window.

## 4. Conclusions

The thermophysical behavior of mono and hybrid water-based nanofluids containing Al_2_O_3_ nanoparticles and PU-NEPCM nanocapsules was comparatively investigated under identical preparation and measurement conditions over 298–313 K. Nine formulations three mono Al_2_O_3_, three mono PU-NEPCM, and three Al_2_O_3_/PU-NEPCM hybrids at total volume fractions of 0.1, 0.3, and 0.5 vol.% were characterized in terms of thermal conductivity, dynamic viscosity, and Day-1 dispersion stability, and were further evaluated using a temperature- and concentration-coupled viscosity correlation, two-factor ANOVA, and synergy and performance indices.

Thermal conductivity increased monotonically with both temperature and concentration, while viscosity increased with particle loading and decreased with temperature. At 0.5 vol.% and 313 K, the hybrid NFH3 yielded an 8.27% thermal conductivity enhancement, exceeding mono Al_2_O_3_ (NFA3, 3.66%) and mono PU-NEPCM (NFPU3, 3.06%) by approximately 4.6 and 5.2 percentage points, respectively. The NFA3 enhancement exceeded the Maxwell effective-medium prediction by ~2.3 percentage points, while NFPU3, for which Maxwell predicts a slight decrease, yielded +3.06%, confirming that the latent heat absorption of the encapsulated n-nonadecane core contributes measurably to the apparent thermal conductivity response. Day-1 zeta potential measurements showed that the hybrid formulations achieved |*ζ*| = 32–37 mV, exceeding the conventional |*ζ*| ≥ 30 mV electrostatic-stabilization threshold and outperforming both mono families at the same total loading. A two-factor ANOVA confirmed that particle concentration was the statistically dominant parameter (*p* < 0.001 in five of six analyses), while temperature became statistically significant only for the hybrid viscosity (*p* = 0.043), consistent with the temperature sensitivity imparted by the encapsulated PCM core. The proposed viscosity correlation captured all three families with *R*^2^ ≥ 0.993 and revealed that the hybrid linear concentration coefficient exceeds the sum of the two mono coefficients, indicating a non-additive viscosity contribution. The synergy and performance index analyses identified NFH1 (0.1 vol.%) as the most balanced formulation, combining strong and consistent synergistic thermal behavior (*SI* = 1.20–1.43) with *PI* ≥ 1 at every temperature; at the highest loading, NFH3 exhibited a pronounced temperature-dependent crossover in *SI* (from 0.85 at 298 K to 1.23 at 313 K) that tracked the n-nonadecane melting interval, providing direct evidence that the synergistic behavior of the hybrid system is activated by the PCM phase transition.

Overall, the results demonstrate that Al_2_O_3_/PU-NEPCM hybrid nanofluids, particularly at low total loading, provide a controlled and balanced thermophysical response under near-ambient operating conditions, combining quantifiable conductivity synergy with a non-degraded viscosity penalty and improved short-term colloidal stability. Because the present performance index captures only the steady-state conductive contribution and does not account for the latent heat storage capacity of the encapsulated PCM core, the effective thermal performance in applications operating across the phase-transition interval is expected to exceed the PI-based assessment reported here. Future work should address long-term colloidal stability under storage and shear conditions, dynamic flow-based heat-transfer performance, and the integration of the latent heat contribution within a unified performance metric for PCM-containing nanofluids.

## Figures and Tables

**Table 1:** Densities of the components used in nanofluid formulation.

Component	Density (g cm^−3^)	Source
Deionized water (25 °C)	0.997	Reference table value
γ-Al_2_O_3_ nanopowder	3.95	Manufacturer specification (Sigma-Aldrich)
PU-NEPCM particles	1.19	Measured (gas pycnometer)

**Table 2:** Nanofluid sample codes and compositions (vol.%).

Sample Code	Nanofluid Type	Total vol.%	Al_2_O_3_ vol.%	PU Nanocapsules vol.%
NF0	Water + surfactant (NP-10, 0.5 wt.%)	—	—	—
NFA1	Al_2_O_3_/water	0.1	0.1	—
NFA2	Al_2_O_3_/water	0.3	0.3	—
NFA3	Al_2_O_3_/water	0.5	0.5	—
NFPU1	PU nanocapsules/water	0.1	—	0.1
NFPU2	PU nanocapsules/water	0.3	—	0.3
NFPU3	PU nanocapsules/water	0.5	—	0.5
NFH1	Al_2_O_3_ + PU nanocapsules/water	0.1	0.05	0.05
NFH2	Al_2_O_3_ + PU nanocapsules/water	0.3	0.15	0.15
NFH3	Al_2_O_3_ + PU nanocapsules/water	0.5	0.25	0.25

**Table 3:** Comparative summary of the morphological, density, and thermal characterization parameters of the PU-NEPCM nanocapsules obtained in this work and in the previous work of the authors [23].

Parameter	This Work	[23]
Mean particle diameter (nm)	120.0 ± 40.7	~103
Particle size range (nm)	63–291	55–175
Number of particles analyzed	703 (automated)	>200 (manual)
Density (g cm^−3^)	1.19	1.19
Pure n-nonadecane *T*_*m* (°C)	30.99	32.12
Pure n-nonadecane Δ*H*_*m* (J g^−1^)	185.92	212.18
Capsule *T*_*m* (°C)	29.65	30.54
Capsule Δ*H*_*m* (J g^−1^)	83.32	92.85
Encapsulation ratio *η* (%)	44.8	44.7

**Table 4:** Coefficients of the temperature- and concentration-coupled viscosity correlation (Equation (5)), fitted to the experimental data of each nanofluid family.

Family	*A*_0_ (mPa s)	*B*_0_ (K)	*C*	*D*	*r* ^2^
NFA (Al_2_O_3_/water)	1.406 × 10^−3^	1931.2	+0.155	−0.069	0.997
NFPU (PU-NEPCM/water)	1.341 × 10^−3^	1945.7	+0.039	+0.055	0.998
NFH (Al_2_O_3_ + PU-NEPCM/water)	1.702 × 10^−3^	1874.1	+0.232	−0.153	0.993

**Table 5:** Summary of ANOVA results for the normalized thermal conductivity and normalized dynamic viscosity of the three nanofluid families. Significance level α = 0.05.

System	Response	*p*-Value (Temperature)	*p*-Value (Concentration)	Dominant Factor	*R*^2^ (%)
NFA	$k_{eff}/k_{bf}$	0.119	<0.001	Concentration	97.67
NFPU	$k_{eff}/k_{bf}$	0.512	<0.001	Concentration	99.12
NFH	$k_{eff}/k_{bf}$	0.059	<0.001	Concentration	96.22
NFA	$\mu_{eff}/\mu_{bf}$	0.199	<0.001	Concentration	93.24
NFPU	$\mu_{eff}/\mu_{bf}$	0.375	0.002	Concentration	88.88
NFH	$\mu_{eff}/\mu_{bf}$	0.043	<0.001	Concentration	93.52

## Data Availability

The data presented in this study are available within the article and its Supplementary Materials. Additional data are available from the corresponding author upon reasonable request.

## Supplementary Materials

The following supporting information can be downloaded at: https://www.mdpi.com/article/10.3390/nano16120746/s1. Section S1: Thermal Conductivity Data; Section S2: Dynamic Viscosity Data; Section S3: Zeta Potential Measurements; Section S5: Maxwell Effective-Medium Predictions; Section S6: Arrhenius Viscosity Correlation; Section S7: Detailed Two-Factor ANOVA Results; Section S8: Complete Synergy and Performance Indices.
