# Supplementary material for: Thermal Conductivity and Dynamic Viscosity of Water-Based Al2O3 and Polyurethane-Nanoencapsulated n-Nonadecane Nanofluids: A Comparative Experimental Study of Mono and Hybrid Formulations
Source: Nanomaterials (Basel). 2026 Jun 15;16(12):746. doi: 10.3390/nano16120746 (PMC13304567; doi:10.3390/nano16120746)
Supplement: Supplementary file 1 [file nanomaterials-16-00746-s001.zip › nanomaterials-4373386-supplementary.pdf]

Supplementary Material

Comparative thermophysical study of water-based mono Al_2O_3, mono PU-NEPCM, and Al_2O_3/PU-NEPCM hybrid nanofluids: thermal conductivity, viscosity, and indicator-based trade-off analysis

Submitted to: Nanomaterials

This document compiles the full numerical data, statistical outputs, and model-based calculations supporting the main manuscript. All section references herein refer to the corresponding sections of the main text unless otherwise specified.

Contents

Section S1: Thermal Conductivity Data S-3
Section S2: Dynamic Viscosity Data S-5
Section S3: Zeta Potential Measurements S-7
Section S5: Maxwell Effective-Medium Predictions S-9
Section S6: Arrhenius Viscosity Correlation S-11
Section S7: Detailed Two-Factor ANOVA Results S-14
Section S8: Complete Synergy and Performance Indices S-17

Note: Section S4 (DSC raw data) is omitted from this supplementary document; the complete DSC characterization of pure n-nonadecane and PU/n-nonadecane nanocapsules is provided in Table 3 of the main text together with the thermogram comparison in Figure 4.

Section S1: Thermal Conductivity Data

This section presents the full mean values and standard deviations of the thermal conductivity measurements that underlie Figure 6 of the main text. Each value corresponds to the average of 15 consecutive readings taken at the target temperature, after rejecting individual readings that lie outside ±2σ of the running mean, as described in Section 2.3.2 of the main text. Standard deviations reported below correspond to the surviving population after outlier rejection. All measurements were performed with the KD2 Pro analyzer and the KS-1 transient hot-wire sensor (Decagon Devices, ±5% accuracy).

Table S1.1. Effective thermal conductivity k_eff (W m^{-1} K^{-1}) of the surfactant-containing base fluid (NF0; 0.5 wt% NP-10 in deionized water). Mean ± 1 SD of 15 replicate readings after 2σ outlier rejection.

Temperature (K)	Mean	SD
298	0.5999	0.0050
303	0.6039	0.0055
308	0.6066	0.0091
313	0.6097	0.0095

Table S1.2. Effective thermal conductivity k_eff (W m^{-1} K^{-1}) of the mono Al_2O_3 family (NFA1–NFA3). Mean ± 1 SD of 15 replicate readings (2σ outlier rejected).

Temperature (K)	NFA1	NFA1	NFA2	NFA2	NFA3	NFA3
	Mean	SD	Mean	SD	Mean	SD
298	0.6123	0.0058	0.6171	0.0033	0.6215	0.0047
303	0.6160	0.0070	0.6197	0.0047	0.6254	0.0050
308	0.6210	0.0062	0.6237	0.0049	0.6296	0.0046
313	0.6250	0.0058	0.6265	0.0057	0.6320	0.0052

Table S1.3. Effective thermal conductivity k_eff (W m^{-1} K^{-1}) of the mono PU-NEPCM family (NFPU1–NFPU3). Mean ± 1 SD of 15 replicate readings (2σ outlier rejected).

Temperature (K)	NFPU1	NFPU1	NFPU2	NFPU2	NFPU3	NFPU3
	Mean	SD	Mean	SD	Mean	SD
298	0.6019	0.0033	0.6106	0.0036	0.6197	0.0042
303	0.6063	0.0043	0.6150	0.0046	0.6236	0.0052
308	0.6101	0.0051	0.6184	0.0056	0.6272	0.0090
313	0.6143	0.0049	0.6212	0.0049	0.6283	0.0048

Table S1.4. Effective thermal conductivity k_eff (W m⁻¹ K⁻¹) of the Al_2O_3/PU-NEPCM hybrid family (NFH1–NFH3). Mean ± 1 SD of 15 replicate readings (2σ outlier rejected).

Temperature (K)	NFH1	NFH1	NFH2	NFH2	NFH3	NFH3
	Mean	SD	Mean	SD	Mean	SD
298	0.6186	0.0065	0.6263	0.0044	0.6350	0.0071
303	0.6246	0.0080	0.6300	0.0055	0.6450	0.0055
308	0.6282	0.0066	0.6345	0.0057	0.6494	0.0080
313	0.6336	0.0091	0.6394	0.0088	0.6601	0.0148

Note on NFH3 at 313 K. The comparatively larger standard deviation observed for NFH3 at 313 K (0.015 W m^{-1} K^{-1}) relative to the other entries (0.003–0.009 W m^{-1} K^{-1}) is consistent with the increased measurement variability expected around the solid–liquid transition of the encapsulated n-nonadecane core (T_m ≈ 30 °C), as the latent heat exchange between the probe vicinity and the surrounding capsules locally perturbs the transient hot-wire response. This observation is in line with the temperature-dependent synergistic enhancement of NFH3 discussed in Sections 3.3 and 3.6 of the main text.

Section S2: Dynamic Viscosity Data

This section presents the full mean values of the dynamic viscosity measurements that underlie Figure 7 of the main text. Three independent readings were collected at each target temperature using the SV-10 sine-wave vibroviscometer (A&D Company, Japan). Because the SV-10 reports viscosity with a resolution of two decimal places, replicate readings frequently yielded identical numerical values; the ±1 % manufacturer-stated accuracy band of the instrument is therefore used in the tables below for the error bar (reported in the "±1 % band" column) rather than the replicate standard deviation. Calibration of the instrument against deionized water yielded agreement within ±1 % across the investigated range (Section 2.3.2 of the main text).

Table S2.1. Dynamic viscosity μ (mPa·s) of the surfactant-containing base fluid (NF0). Mean of three replicate readings; ±1 % band corresponds to the SV-10 manufacturer-stated accuracy.

Temperature (K)	Mean	± 1 % band
298	0.920	0.0092
303	0.826	0.0083
308	0.743	0.0074
313	0.667	0.0067

Table S2.2. Dynamic viscosity μ (mPa·s) of the mono Al_2O_3 family (NFA1–NFA3). Mean of three replicate readings; ±1 % band per SV-10 accuracy.

Temperature (K)	NFA1	NFA1	NFA2	NFA2	NFA3	NFA3
	Mean	± 1 %	Mean	± 1 %	Mean	± 1 %
298	0.933	0.0093	0.950	0.0095	0.960	0.0096
303	0.840	0.0084	0.860	0.0086	0.886	0.0089
308	0.753	0.0075	0.773	0.0077	0.793	0.0079
313	0.676	0.0068	0.703	0.0070	0.710	0.0071

Table S2.3. Dynamic viscosity μ (mPa·s) of the mono PU-NEPCM family (NFPU1–NFPU3). Mean of three replicate readings; ±1 % band per SV-10 accuracy.

Temperature (K)	NFPU1	NFPU1	NFPU2	NFPU2	NFPU3	NFPU3
	Mean	± 1 %	Mean	± 1 %	Mean	± 1 %
298	0.926	0.0093	0.933	0.0093	0.940	0.0094
303	0.830	0.0083	0.840	0.0084	0.853	0.0085
308	0.747	0.0075	0.753	0.0075	0.776	0.0078
313	0.670	0.0067	0.683	0.0068	0.696	0.0070

Table S2.4. Dynamic viscosity μ (mPa·s) of the Al_2O_3/PU-NEPCM hybrid family (NFH1–NFH3). Mean of three replicate readings; ±1 % band per SV-10 accuracy.

Temperature (K)	NFH1	NFH1	NFH2	NFH2	NFH3	NFH3
	Mean	± 1 %	Mean	± 1 %	Mean	± 1 %
298	0.943	0.0094	0.960	0.0096	0.970	0.0097
303	0.850	0.0085	0.873	0.0087	0.900	0.0090
308	0.773	0.0077	0.790	0.0079	0.820	0.0082
313	0.686	0.0069	0.710	0.0071	0.730	0.0073

Note. Across both datasets, viscosity decreases monotonically with temperature while increasing with total solid volume fraction for all three families; thermal conductivity increases with both temperature and concentration. The hybrid suspensions (NFH1–NFH3) consistently show the largest absolute thermal-conductivity values at every temperature and the largest viscosity penalty at every concentration, in line with the trends reported in Sections 3.3 and 3.4 of the main text.

Section S3: Zeta Potential Measurements

The colloidal stability of the nine freshly prepared nanofluids was characterized on Day 1 by zeta potential measurements at 25 °C using a Malvern Panalytical Zetasizer Nano in a clear disposable zeta cell (dispersant: deionized water; RI = 1.330; viscosity = 0.8872 cP; dielectric constant = 78.5). For each sample, three independent measurements were performed, each consisting of 12 zeta runs. The mean zeta potential values and the corresponding standard deviations reported by the Zetasizer software for the primary peak of each distribution are summarized in Table S3.1. The result quality returned by the Zetasizer software was "Good" for all 27 individual measurements.

Table S3.1. Day-1 zeta potential of the nine prepared nanofluids as reported by the Zetasizer Nano software. Mean and SD values correspond to the primary peak of the zeta potential distribution (100 % area in all cases), derived from 12 zeta runs per measurement, with three independent measurements per sample. All measurements at 25.0 °C, in deionized water as dispersant, with no dilution applied.

Sample	ζ (mV)	SD (mV)	|ζ| (mV)	Quality
NFA1	−12.3	2.81	12.3	Good
NFA2	−14.6	3.96	14.6	Good
NFA3	−17.6	7.46	17.6	Good
NFPU1	−13.9	7.07	13.9	Good
NFPU2	−18.6	6.76	18.6	Good
NFPU3	−26.0	5.11	26.0	Good
NFH1	−32.1	4.61	32.1	Good
NFH2	−34.0	4.24	34.0	Good
NFH3	−37.0	4.54	37.0	Good

Note on SD reporting. The reported standard deviation corresponds to the width of the zeta potential distribution within each measurement as output by the Zetasizer software for the primary peak, characterizing the polydispersity of the suspended population. Inter-measurement reproducibility across the three replicates was within ±2 mV for all samples, supporting the use of these data for the comparative Day-1 stability assessment in Section 3.2 of the main text.

Stability classification. Across the three families, the zeta potential magnitude |ζ| increased systematically from mono Al_2O_3 (NFA: 12.3–17.6 mV) through mono PU-NEPCM (NFPU: 13.9–26.0 mV) to the Al_2O_3/PU-NEPCM hybrids (NFH: 32.1–37.0 mV). All three hybrid formulations exceeded the conventionally accepted $|\zeta| \geq 30$ mV threshold for good electrostatic stabilization, in contrast to the mono families. This trend supports the interpretation given in Section 3.2 of the main text that the simultaneous presence of two chemically dissimilar dispersed phases (oxide nanoparticles and polymer-shelled PCM nanocapsules) promotes a more strongly charged interfacial structure than either mono-component system, providing an independent stability baseline for the comparative thermophysical analysis reported in Sections 3.3–3.6.

Instrument: Malvern Panalytical Zetasizer Nano; clear disposable zeta cell; measurement position 2.00 mm; attenuator setting selected automatically; count rate 150–200 kcps, depending on sample; conductivity 0.030–0.040 mS/cm; no dilution applied prior to measurement.

Section S5: Maxwell Effective-Medium Predictions

The measured thermal-conductivity enhancement ratios k_eff/k_bf of all nine nanofluids are compared in this section against the classical Maxwell effective-medium prediction for non-interacting spherical inclusions:

$$k\_eff / k\_bf = [k\_p + 2 \cdot k\_bf + 2 \cdot \varphi \cdot (k\_p - k\_bf)] / [k\_p + 2 \cdot k\_bf - \varphi \cdot (k\_p - k\_bf)] \quad (S5.1)$$

where k_p is the thermal conductivity of the dispersed phase and φ is its total volume fraction. The following inputs were used:

- $k(Al_2O_3) = 30.0\ W\ m^{-1}\ K^{-1}$ (bulk γ-Al_2O_3)
- $k(PU\text{-}NEPCM) = 0.25\ W\ m^{-1}\ K^{-1}$ (bulk, from reference [23])
- k(hybrid, 50:50 v/v) = $15.13\ W\ m^{-1}\ K^{-1}$ (volume-weighted average of the two components)
- k_bf taken from the measured NF0 values at each temperature (Section S1)
- φ taken as the total dispersed-phase volume fraction (0.001, 0.003, 0.005)

Table S5.1. Comparison of measured (k_eff/k_bf)_meas with Maxwell predictions (k_eff/k_bf)_Maxwell for all nine nanofluids at the four investigated temperatures. Δ = (measured − predicted) × 100 (in percentage points).

Sample	φ (vol.%)	T (K)	(k/k_bf)_meas	(k/k_bf)_Maxwell	Δ (pp)
NFA1	0.1	298	1.0207	1.0028	+1.78
NFA1	0.1	303	1.0200	1.0028	+1.72
NFA1	0.1	308	1.0237	1.0028	+2.09
NFA1	0.1	313	1.0251	1.0028	+2.23
NFA2	0.3	298	1.0287	1.0085	+2.02
NFA2	0.3	303	1.0262	1.0085	+1.77
NFA2	0.3	308	1.0282	1.0085	+1.97
NFA2	0.3	313	1.0276	1.0085	+1.91
NFA3	0.5	298	1.0360	1.0142	+2.18
NFA3	0.5	303	1.0356	1.0142	+2.14
NFA3	0.5	308	1.0379	1.0142	+2.37
NFA3	0.5	313	1.0366	1.0142	+2.24
NFPU1	0.1	298	1.0033	0.9993	+0.41
NFPU1	0.1	303	1.0040	0.9993	+0.47
NFPU1	0.1	308	1.0058	0.9993	+0.65
NFPU1	0.1	313	1.0075	0.9993	+0.83
NFPU2	0.3	298	1.0178	0.9978	+2.00
NFPU2	0.3	303	1.0184	0.9978	+2.06
NFPU2	0.3	308	1.0195	0.9978	+2.16
NFPU2	0.3	313	1.0189	0.9978	+2.11
NFPU3	0.5	298	1.0330	0.9964	+3.66

NFPU3	0.5	303	1.0326	0.9964	+3.63
NFPU3	0.5	308	1.0340	0.9963	+3.76
NFPU3	0.5	313	1.0305	0.9963	+3.42
NFH1	0.1	298	1.0312	1.0027	+2.85
NFH1	0.1	303	1.0343	1.0027	+3.16
NFH1	0.1	308	1.0356	1.0027	+3.29
NFH1	0.1	313	1.0392	1.0027	+3.65
NFH2	0.3	298	1.0440	1.0080	+3.60
NFH2	0.3	303	1.0432	1.0080	+3.52
NFH2	0.3	308	1.0460	1.0080	+3.80
NFH2	0.3	313	1.0487	1.0080	+4.07
NFH3	0.5	298	1.0585	1.0134	+4.51
NFH3	0.5	303	1.0681	1.0134	+5.47
NFH3	0.5	308	1.0706	1.0134	+5.72
NFH3	0.5	313	1.0827	1.0134	+6.93

Interpretation. For the mono Al_2O_3 family (NFA), the measured enhancement ratios exceed the Maxwell prediction by approximately +1.7 to +2.4 percentage points across all temperatures and concentrations, consistent with the "anomalous" enhancement widely attributed in the nanofluid literature to Brownian-motion-driven micro-convection and to the formation of an interfacial nanolayer of ordered solvent molecules around the oxide nanoparticles [25, 26]. For the mono PU-NEPCM family (NFPU), the Maxwell model based on the bulk PU-NEPCM conductivity predicts a slight decrease in k_eff (ratios <1) because the polymer-shelled capsules are effectively a low-conductivity dispersed phase; the measured ratios, however, show a positive enhancement of up to +3.8 percentage points, confirming that the latent heat contribution of the encapsulated n-nonadecane core within the 298–313 K phase-transition window provides the dominant enhancement mechanism for this family. For the hybrid family (NFH), the measured enhancement exceeds the Maxwell prediction by +2.9 to +6.9 percentage points, the largest deviation of the three families, and the deviation grows systematically with temperature for the most concentrated sample (NFH3: +4.5 pp at 298 K → +6.9 pp at 313 K). This temperature dependence supports the interpretation given in Section 3.3 of the main text that the coupling between Brownian-driven micro-mixing of the Al_2O_3 nanoparticles and the latent heat exchange of the PU-NEPCM cores becomes progressively more effective as the operating temperature traverses the melting interval of n-nonadecane.

Section S6: Arrhenius Viscosity Correlation

The measured dynamic viscosity values of the three nanofluid families were fitted to the two-factor Arrhenius-type correlation introduced in Section 3.4 of the main text:

$$\mu = (1 + C\cdot\varphi + D\cdot\varphi^2) \cdot A_0 \cdot exp(B_0 / T) \quad (S6.1)$$

where φ is expressed in vol.% (0.1, 0.3, 0.5), T in K, μ in mPa·s, and A_0, B_0, C, D are family-specific fitting coefficients determined by nonlinear least-squares regression on the 12 data points per family (4 temperatures × 3 concentrations). The fitted coefficients reported in Table 4 of the main text were used to generate the family-wise predictions tabulated below; the corresponding goodness-of-fit metrics are summarized in Table S6.1.

Table S6.1. Goodness-of-fit metrics for the Arrhenius correlation [Eq. (S6.1)] applied to each of the three nanofluid families, using the coefficients reported in Table 4 of the main text. r^2 = coefficient of determination on 12 datapoints (4 T × 3 φ); max dev = maximum relative deviation between fitted and measured viscosity, in %.

Family	A_0 (mPa·s)	B_0 (K)	C	D	r^2	Max dev (%)
NFA	1.406e-03	1931.2	+0.155	-0.069	0.9960	1.38
NFPU	1.341e-03	1945.7	+0.039	+0.055	0.9981	1.08
NFH	1.702e-03	1874.1	+0.232	-0.153	0.9912	1.85

Table S6.2. Measured (μ_meas) and Arrhenius-fitted (μ_fit) viscosity values, their absolute residuals (μ_meas − μ_fit), and relative deviation (%) for the NFA family using the coefficients of Table S6.1.

Sample	φ (vol.%)	T (K)	μ_meas (mPa·s)	μ_fit (mPa·s)	Residual	Dev (%)
NFA1	0.1	298	0.9330	0.9307	+0.0023	+0.24
NFA1	0.1	303	0.8400	0.8364	+0.0036	+0.43
NFA1	0.1	308	0.7530	0.7541	-0.0011	-0.15
NFA1	0.1	313	0.6760	0.6823	-0.0063	-0.93
NFA2	0.3	298	0.9500	0.9541	-0.0041	-0.43
NFA2	0.3	303	0.8600	0.8573	+0.0027	+0.31
NFA2	0.3	308	0.7730	0.7731	-0.0001	-0.01
NFA2	0.3	313	0.7030	0.6994	+0.0036	+0.51
NFA3	0.5	298	0.9600	0.9724	-0.0124	-1.29
NFA3	0.5	303	0.8860	0.8738	+0.0122	+1.38
NFA3	0.5	308	0.7930	0.7879	+0.0051	+0.64
NFA3	0.5	313	0.7100	0.7128	-0.0028	-0.40

Table S6.3. Measured (μ_meas) and Arrhenius-fitted (μ_fit) viscosity values, their absolute residuals (μ_meas − μ_fit), and relative deviation (%) for the NFPU family using the coefficients of Table S6.1.

Sample	φ (vol.%)	T (K)	μ_meas (mPa·s)	μ_fit (mPa·s)	Residual	Dev (%)
NFPU1	0.1	298	0.9260	0.9225	+0.0035	+0.38
NFPU1	0.1	303	0.8300	0.8282	+0.0018	+0.21
NFPU1	0.1	308	0.7470	0.7463	+0.0007	+0.10
NFPU1	0.1	313	0.6700	0.6746	-0.0046	-0.69
NFPU2	0.3	298	0.9330	0.9337	-0.0007	-0.07
NFPU2	0.3	303	0.8400	0.8383	+0.0017	+0.20
NFPU2	0.3	308	0.7530	0.7553	-0.0023	-0.31
NFPU2	0.3	313	0.6830	0.6828	+0.0002	+0.03
NFPU3	0.5	298	0.9400	0.9489	-0.0089	-0.95
NFPU3	0.5	303	0.8530	0.8520	+0.0010	+0.12
NFPU3	0.5	308	0.7760	0.7676	+0.0084	+1.08
NFPU3	0.5	313	0.6960	0.6940	+0.0020	+0.29

Table S6.4. Measured (μ_meas) and Arrhenius-fitted (μ_fit) viscosity values, their absolute residuals (μ_meas − μ_fit), and relative deviation (%) for the NFH family using the coefficients of Table S6.1.

Sample	φ (vol.%)	T (K)	μ_meas (mPa·s)	μ_fit (mPa·s)	Residual	Dev (%)
NFH1	0.1	298	0.9430	0.9365	+0.0065	+0.69
NFH1	0.1	303	0.8500	0.8442	+0.0058	+0.68
NFH1	0.1	308	0.7730	0.7636	+0.0094	+1.22
NFH1	0.1	313	0.6860	0.6928	-0.0068	-1.00
NFH2	0.3	298	0.9600	0.9678	-0.0078	-0.82
NFH2	0.3	303	0.8730	0.8724	+0.0006	+0.07
NFH2	0.3	308	0.7900	0.7891	+0.0009	+0.12
NFH2	0.3	313	0.7100	0.7160	-0.0060	-0.84
NFH3	0.5	298	0.9700	0.9879	-0.0179	-1.85
NFH3	0.5	303	0.9000	0.8905	+0.0095	+1.05
NFH3	0.5	308	0.8200	0.8055	+0.0145	+1.77
NFH3	0.5	313	0.7300	0.7309	-0.0009	-0.12

Interpretation. Across all three families, the correlation captures the experimental viscosity within ±1.85 % (max), well below the ±5 % accuracy quoted in most engineering nanofluid correlations and comparable to the ±1 % manufacturer-stated accuracy of the SV-10 vibroviscometer. The temperature coefficient B_0 (1874–1946 K) is similar across the three families, corresponding to an apparent flow activation energy of ~15.6–16.2 kJ mol^{-1} and indicating that the base fluid dominates the temperature dependence. The linear concentration coefficient C varies substantially:

C(NFPU) = +0.039 < C(NFA) = +0.155 < C(NFH) = +0.232. Notably, C(NFH) exceeds the sum of the two mono coefficients (C(NFA) + C(NFPU) ≈ 0.194) by approximately 20 %, providing quantitative evidence for the non-additive contribution to the hybrid viscosity response discussed in Section 3.4 of the main text. The negative quadratic coefficient D(NFH) = −0.153 indicates a saturating behavior between 0.3 and 0.5 vol.%, consistent with the onset of inter-particle hindrance effects at the upper end of the investigated concentration window.

Section S7: Detailed Two-Factor ANOVA Results

A two-factor analysis of variance (ANOVA) was performed using Minitab v18 on the normalized thermal conductivity (k/k_bf) and normalized dynamic viscosity (μ/μ_bf) of each of the three nanofluid families (NFA, NFPU, NFH). Temperature (4 levels: 298, 303, 308, 313 K) and total volume fraction (3 levels: 0.1, 0.3, 0.5 vol.%) were used as the independent factors with the (−1, 0, +1) factor coding scheme. The detailed source-of-variation tables, including degrees of freedom (DF), adjusted sums of squares (Adj SS), adjusted mean squares (Adj MS), F-statistics, and p-values, are presented below. The corresponding model summary statistics (S, R^2, adjusted R^2, and predicted R^2) are reported beneath each table.

Table S7.1. Two-factor ANOVA for the normalized thermal conductivity (k_eff/k_bf) of the mono Al_2O_3 family (NFA).

Source	DF	Adj SS	Adj MS	F-Value	P-Value
Temperature (K)	3	1.5×10^{-5}	5.0×10^{-6}	2.97	0.119
Concentration (vol. %)	2	4.08×10^{-4}	2.04×10^{-4}	121.36	<0.001
Error	6	1.0×10^{-5}	1.7×10^{-6}	—	—
Total	11	4.33×10^{-4}	—	—	—

Model summary: S = 1.297×10^{-3}, R^2 = 97.67 %, R^2(adj) = 95.73 %, R^2(pred) = 90.68 %.

Table S7.2. Two-factor ANOVA for the normalized dynamic viscosity (μ_eff/μ_bf) of the mono Al_2O_3 family (NFA).

Source	DF	Adj SS	Adj MS	F-Value	P-Value
Temperature (K)	3	3.80×10^{-4}	1.27×10^{-4}	2.12	0.199
Concentration (vol. %)	2	4.562×10^{-3}	2.281×10^{-3}	38.22	<0.001
Error	6	3.58×10^{-4}	6.0×10^{-5}	—	—
Total	11	5.300×10^{-3}	—	—	—

Model summary: S = 7.725×10^{-3}, R^2 = 93.24 %, R^2(adj) = 87.61 %, R^2(pred) = 72.98 %.

Table S7.3. Two-factor ANOVA for the normalized thermal conductivity (k_eff/k_bf) of the mono PU-NEPCM family (NFPU).

Source	DF	Adj SS	Adj MS	F-Value	P-Value
Temperature (K)	3	6.0×10^{-6}	2.0×10^{-6}	0.86	0.512
Concentration (vol. %)	2	1.500×10^{-3}	7.50×10^{-4}	336.39	<0.001
Error	6	1.3×10^{-5}	2.2×10^{-6}	—	—
Total	11	1.519×10^{-3}	—	—	—

Model summary: S = 1.493×10^{-3}, R^2 = 99.12 %, R^2(adj) = 98.39 %, R^2(pred) = 96.48 %.

Table S7.4. Two-factor ANOVA for the normalized dynamic viscosity (μ_eff/μ_bf) of the mono PU-NEPCM family (NFPU).

Source	DF	Adj SS	Adj MS	F-Value	P-Value
Temperature (K)	3	1.58×10^{-4}	5.3×10^{-5}	1.24	0.375
Concentration (vol. %)	2	1.877×10^{-3}	9.38×10^{-4}	22.11	0.002
Error	6	2.55×10^{-4}	4.2×10^{-5}	—	—
Total	11	2.289×10^{-3}	—	—	—

Model summary: S = 6.515×10^{-3}, R^2 = 88.88 %, R^2(adj) = 79.61 %, R^2(pred) = 55.50 %.

Table S7.5. Two-factor ANOVA for the normalized thermal conductivity (k_eff/k_bf) of the Al_2O_3/PU-NEPCM hybrid family (NFH).

Source	DF	Adj SS	Adj MS	F-Value	P-Value
Temperature (K)	3	2.41×10^{-4}	8.0×10^{-5}	4.37	0.059
Concentration (vol. %)	2	2.562×10^{-3}	1.281×10^{-3}	69.80	<0.001
Error	6	1.10×10^{-4}	1.8×10^{-5}	—	—
Total	11	2.913×10^{-3}	—	—	—

Model summary: S = 4.284×10^{-3}, R^2 = 96.22 %, R^2(adj) = 93.07 %, R^2(pred) = 84.88 %.

Table S7.6. Two-factor ANOVA for the normalized dynamic viscosity (μ_eff/μ_bf) of the Al_2O_3/PU-NEPCM hybrid family (NFH).

Source	DF	Adj SS	Adj MS	F-Value	P-Value
Temperature (K)	3	1.302×10^{-3}	4.34×10^{-4}	5.15	0.043
Concentration (vol. %)	2	6.004×10^{-3}	3.002×10^{-3}	35.61	<0.001
Error	6	5.06×10^{-4}	8.4×10^{-5}	—	—
Total	11	7.812×10^{-3}	—	—	—

Model summary: S = 9.182×10^{-3}, R^2 = 93.52 %, R^2(adj) = 88.13 %, R^2(pred) = 74.10 %.

Note. Among the six analyses, the temperature factor reached statistical significance ($p < 0.05$) only for the hybrid viscosity (Table S7.6, $p = 0.043$), consistent with the temperature sensitivity imparted to the NFH suspensions by the solid–liquid transition of the encapsulated n-nonadecane core within the 298–313 K window. Particle concentration was the dominant factor across all six analyses ($p < 0.001$ in five cases, $p = 0.002$ in the sixth), confirming the qualitative trends discussed in Section 3 of the main text.

Section S8: Complete Synergy and Performance Indices

This section presents the full numerical values of the synergy index (SI) and performance index (PI) introduced in Section 2.4 of the main text. These data underlie Figure 8 of the main text.

$$SI = (k\_hyb/k\_bf - 1) / [(k\_A/k\_bf - 1) + (k\_PU/k\_bf - 1)] \quad (S8.1)$$

The synergy index quantifies the departure of the hybrid response from a linear-additive baseline reconstructed from the two corresponding mono-component nanofluids. SI > 1 indicates synergistic behavior (hybrid exceeds the sum of the mono contributions), SI = 1 indicates additive behavior, and SI < 1 indicates sub-additive behavior.

Table S8.1. Synergy index (SI) of the three hybrid concentrations (NFH1: 0.1 vol.%, NFH2: 0.3 vol.%, NFH3: 0.5 vol.%) at the four investigated temperatures. Values calculated from Eq. (S8.1) using the measured thermal conductivities of all nine nanofluids and the NF0 base fluid.

Hybrid sample	φ (vol.%)	SI @ 298 K	SI @ 303 K	SI @ 308 K	SI @ 313 K
NFH1	0.1	1.299	1.428	1.207	1.201
NFH2	0.3	0.946	0.970	0.965	1.049
NFH3	0.5	0.848	0.998	0.982	1.232

Observations. The lowest-loaded hybrid (NFH1, 0.1 vol.%) exhibits consistently synergistic behavior (SI = 1.20–1.43) across the entire investigated temperature range, with the strongest synergy at the lower temperatures (below the n-nonadecane melting interval). At the intermediate loading (NFH2, 0.3 vol.%), the response is essentially additive (SI ≈ 0.95–1.05). At the highest loading (NFH3, 0.5 vol.%), the synergy index shows a temperature-dependent crossover from sub-additive (SI ≈ 0.85 at 298 K) to synergistic (SI ≈ 1.23 at 313 K), reflecting the increasing dominance of latent heat contributions as the operating temperature traverses the PCM melting interval. The strongest single observation is NFH1 at 303 K (SI = 1.43), corresponding to a hybrid response that exceeds the linear additive baseline by 43 %. These patterns are discussed in detail in Section 3.6 of the main text.

$$PI = (k\_nf/k\_bf) / (\mu\_nf/\mu\_bf) \quad (S8.2)$$

The performance index quantifies the net practical balance between thermal conductivity enhancement and viscosity penalty. PI > 1 indicates a net favorable trade-off (steady-state conductive contribution exceeds the viscous penalty), PI = 1 indicates neutral balance, and PI < 1 indicates a net unfavorable steady-state response. As noted in Section 2.4 of the main text, PI does not capture the latent heat storage capacity of the NEPCM particles and should therefore be interpreted as a lower bound on the practical thermal management benefit for PCM-containing systems.

Table S8.2. Performance index (PI) of all nine nanofluids at the four investigated temperatures. Values calculated from Eq. (S8.2) using the measured thermal conductivity and viscosity ratios.

Sample	φ (vol.%)	PI @ 298 K	PI @ 303 K	PI @ 308 K	PI @ 313 K
NFA1	0.1	1.0064	1.0030	1.0101	1.0114
NFA2	0.3	0.9962	0.9856	0.9883	0.9749
NFA3	0.5	0.9928	0.9655	0.9725	0.9738
NFPU1	0.1	0.9968	0.9991	1.0004	1.0030

NFPU2	0.3	1.0037	1.0014	1.0059	0.9950
NFPU3	0.5	1.0110	0.9999	0.9900	0.9876
NFH1	0.1	1.0060	1.0051	0.9954	1.0104
NFH2	0.3	1.0005	0.9871	0.9838	0.9852
NFH3	0.5	1.0039	0.9802	0.9700	0.9892

Observations. The performance index of all nine nanofluids lies within a narrow band between approximately 0.97 and 1.01 across the investigated temperature range, reflecting the small absolute differences in normalized properties at the low loadings considered here. Among the mono families, the lowest-loaded mono Al_2O_3 (NFA1) shows the largest PI values (1.003–1.011), while the more heavily loaded samples show PI values slightly below unity due to the larger viscosity penalty. The lowest-loaded hybrid (NFH1) shows consistently favorable PI values (0.995–1.010) and combines this neutral-to-favorable steady-state balance with the strongest thermal-conductivity synergy (SI = 1.20–1.43, Table S8.1) and the highest Day-1 colloidal stability ($|\zeta|$ = 32.1 mV, Section S3), supporting the identification of NFH1 in Section 3.6 of the main text as the most balanced candidate for near-ambient liquid-based thermal-management applications.

Table S8.3. Combined summary of the key indicator-based metrics at 313 K and the highest investigated loading (0.5 vol.%) for each family, illustrating the overall trade-off captured by SI, PI, and the underlying normalized properties.

Sample	k/k_bf	μ/μ_bf	PI	SI	$|\zeta|$ (mV)
NFA3	1.037	1.064	0.974	—	17.6
NFPU3	1.031	1.043	0.988	—	26.0
NFH3	1.083	1.094	0.989	1.232	37.0

— End of Supplementary Material —
